# Simultaneous Quantification of *γ*-Hydroxybutyrate, *γ*-Butyrolactone, and 1,4-Butanediol in Four Kinds of Beverages

**DOI:** 10.1155/2020/8837743

**Published:** 2020-07-15

**Authors:** Shaoming Jin, Xiao Ning, Jin Cao, Yaonan Wang

**Affiliations:** ^1^National Institutes for Food and Drug Control, 100050 Beijing, China; ^2^Core Facilities Centre, Capital Medical University, 100069 Beijing, China

## Abstract

*γ*-Hydroxybutyrate (GHB) is a neurotransmitter, which exhibits a strong central nervous system depressant effect. The abuse of GHB or its precursor substances (*γ*-butyrolactone (GBL) and 1,4-butanediol (1,4-BD)) may cause serious problems. This study developed a fast and effective UHPLC-MS/MS method for the simultaneous quantification of GHB, GBL, and 1,4-BD in four popular beverages, including carbonated drinks, tea, apple cider vinegar, and coffee. The established method overcomes the influence of the in-source collision-induced dissociation of unstable compounds during quantification. The limits of detection were 0.2 *μ*g/mL for GBL and 0.5 *μ*g/mL for GHB and 1,4-BD with excellent linearity in the range of 0.2–50 *μ*g/mL. The recoveries of the three compounds at three spiked levels (2.5, 5.0, and 10.0 *μ*g/mL) in the four kinds of beverages studied were between 90 and 110%, while the relative standard deviations (RSDs) were all <10%. The matrix effect was negligible using this simple and appropriate preprocessed procedure. The method established in this study can quickly and reliably detect the GHB content and its analogues in beverages.

## 1. Introduction

*γ*-Hydroxybutyrate (GHB) is a neurotransmitter, which exhibits a strong central nervous system depressant effect [1]. GHB has been used as a clinical anesthetic since the 1960s. However, the therapeutic concentration range of GHB is limited, and it is dangerous when mixed with alcohol or other central nervous system sedatives [2]. The intense sedative and amnesic effect combined with its colorless and tasteless characteristics allow GHB to be easily used by criminals to engage in illegal activities. Therefore, GHB is listed in as a controlled substance. GHB abuse has been reported in drug-facilitated sexual assaults (DFSA), such as robberies, sexual assaults, and fraudulent gambling [3]. Recently, the illicit use of GHB has become a serious social problem. GHB is most commonly available from street markets or over the Internet and can be taken as a colorless, odorless liquid or white powder tablets.

**Figure 1:**
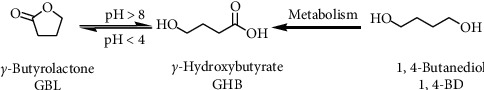
The chemical structures and transformation of GBL, GHB, and 1,4-BD.

*γ*-Butyrolactone (GBL) and 1,4-butanediol (1,4-BD) are two GHB precursors [4, 5]. The chemical structures of these compounds are shown in Figure 1. GBL and GHB are transformed into each other upon changing the pH. GHB can be converted into GBL under acidic conditions (pH < 4), whereas GBL is converted into GHB under alkaline conditions (pH > 8). 1,4-BD can be transformed into GHB via its metabolism in the human body and is another precursor that is dangerous as GHB [6–8].

Beverages containing illegally added GHB are always found in recreational places. In addition to strengthening the supervision of GHB itself, attention should also be paid toward GBL and 1,4-BD. Most of the existing detection methods are aimed at the detection of GHB in biological samples, such as plasma and urine [9]. The types of methods used also vary and include chemical coloration [10], gas chromatography, gas chromatography-tandem mass spectrometry (GC-MS) [11], capillary electrophoresis, liquid chromatography [12, 13], and liquid chromatography-tandem mass spectrometry (LC-MS or LC-MS/MS) [14–18]. Among these methods, LC-MS/MS has a powerful quantitative ability, which can be used for the simultaneous quantification of the three target compounds in drinks [19]. The sample preparation methods used for LC-MS/MS are always simple and do not require derivatization.

In this study, a rapid and accurate UHPLC-MS/MS method for the determination of GHB, GBL, and 1,4-BD in beverages has been developed. In order to prove the universality of the method, four kinds of beverages were chosen with different matrixes, namely, carbonated drinks, tea, apple cider vinegar, and coffee. The sensitivity, linearity, accuracy, and repeatability were characterized to validate the method's performance.

## 2. Materials and Methods

### 2.1. Chemicals and Reagents

GHB, GBL, and 1,4-BD were purchased from Sigma-Aldrich Chemical Co. (St. Louis, MO, USA). Methanol was purchased from Fisher Chemical Co. Formic acid suitable for analysis was obtained from Merck Co. (Darmstadt, Germany). Pure water was laboratory made. All the beverages were purchased from a local supermarket (Beijing, China).

### 2.2. Instrumentation

The LC-MS/MS system consisted of an Agilent 1290 UHPLC (Agilent Technologies Co., Santa Clara, CA, USA) coupled with an Agilent 6460 triple quadrupole mass spectrometer. The LC-MS/MS system was controlled using a Mass Hunter workstation (B.07.00). An HSS T3 (3.0 × 150 mm, 1.8 *μ*m, Waters, Milford, MA, USA) UHPLC column was used for the separation. A KQ 2200E ultrasonic cleaner (Shanghai Kunshan Co.) was used to remove any excess gas from the carbonated drinks. All precision balances were obtained from Mettler-Toledo International Inc.

### 2.3. Preparation of the Calibrants

The mixed working solution was prepared by diluting a stock solution containing three compounds at a concentration of 500 mg/L. The concentration of the working solution was 100 mg/L, and the concentrations of the calibrants were prepared at 0.2, 2.0, 5.0, 10.0, 20.0, and 50.0 *μ*g/mL from the working solution via a stepwise dilution method.

### 2.4. Preparation of the Samples

Before testing, the samples were preprocessed using different methods. For carbonated drinks, the samples were subjected to ultrasonication for 20 min to remove the excess gas; for tea, the samples were subjected to centrifugation (15000 rpm, 10 min) to remove any insoluble solids; for apple cider vinegar, the pH of the sample was adjusted to pH neutral using a 10% aqueous ammonia solution; and for coffee, the sample was filtered to remove any insoluble matter. All the samples were filtered using a 0.22 *μ*m filter membrane prior to injection into the LC-MS/MS system.

### 2.5. UHPLC Separation and Mass Conditions

Water (A) and methanol (B) containing 0.2% formic acid (*v*/*v*) were used as mobile phases. An HSS T3 (3.0 × 150 mm, 1.7 *μ*m, waters) column was used for the separation. Although the separation was carried out in 5% phase B, in order to reduce the accumulation of the low polarity components in the samples on the chromatographic column, a gradient flush condition was set as follows: 0 min = 5% B, 4.5 min = 5% B, 5.5 min = 95% B, 9.5 min = 95% B, 9.6 min = 5% B; and the whole analysis time was 12 min. The flow rate was 0.3 mL/min, and the column temperature was set at 40°C. All three compounds were analyzed using an electrospray ionization source in the positive mode at a capillary voltage of 3500 V, nebulizer gas pressure of 35 psi, and dry gas flow rate of 7 L/min at 350°C.

## 3. Results and Discussion

### 3.1. Method Optimization

#### 3.1.1. Mass Spectrometric Detection

**Figure 2:**
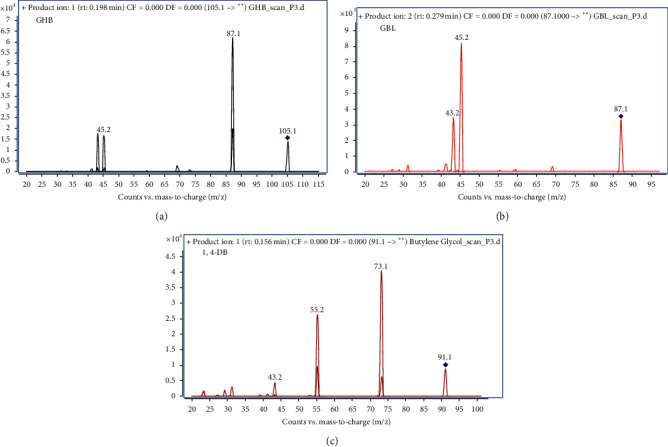
The product ion spectra obtained for GHB (a), GBL (b), and 1,4-BD (c).

Compound optimizations were performed via the direct injection of GHB, GBL, and 1,4-BD into the ESI source in both positive and negative modes. These compounds can be detected in both ion modes [9, 20–24], but the response in the positive mode was better than that observed in the negative mode. Consequently, the positive mode was chosen as the optimal ionization mode. In the positive ionization mode, the protonated quasimolecular ions of GHB, GBL, and 1,4-DB were observed at *m*/*z* 105.1, 87.1, and 91.1, respectively. The product ion spectra obtained for these compounds are shown in Figure 2. The *m*/*z* peaks observed at 87.1 for GHB, 45.2 for GBL, and 73.2 for 1,4-BD exhibited the highest response in their respective MS/MS spectra and were selected as the quantitative ions for their corresponding compounds. The *m*/*z* peaks observed at 45.2 for GHB, 43.2 for GBL, and 1,4-BD were the second-best responding ions and were selected as the qualitative ions.

**Figure 3:**
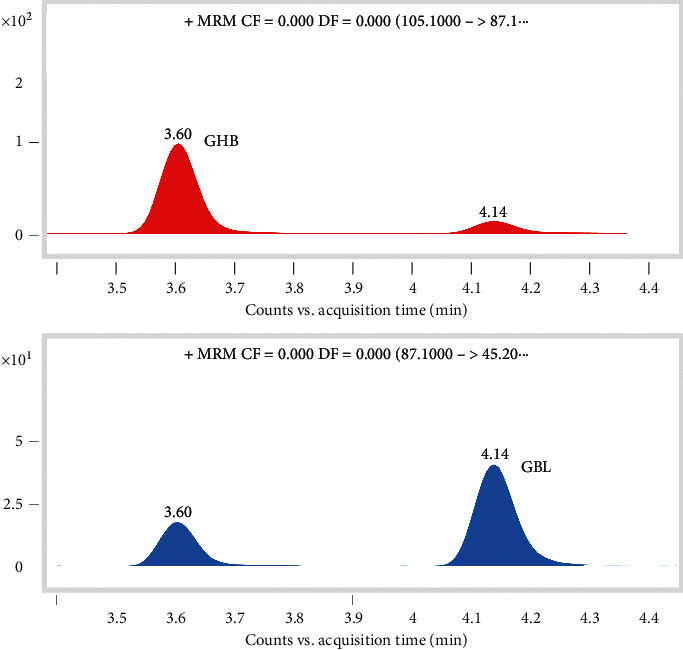
The EICs obtained for GHB and GBL. The retention times for GHB and GBL were 3.60 and 4.14 min, respectively.

Figure 2 shows that the peak observed at *m*/*z* 87.1 was the product ion of GHB and the precursor ion of GBL. This was attributed to GHB losing a water molecule within the instrument ion source, leading to the formation of GBL. In addition, GBL can gain one molecule of water and protonated to form the same *m*/*z* of GHB. In this case, the chromatographic separation of these two compounds was necessary in order to avoid their coelution and eliminate the interference between them in the quantitative results. Their corresponding extraction ion chromatograms (EIC) are shown in Figure 3. It is of interest that the method can distinguish between the in-source generated GBL or (GHB − H_2_O + H)^+^ and actual GBL in one sample. The peak resolution observed for GHB and GBL calculated from Figure 3 was 5.4, which indicated the complete separation of these two compounds.

#### 3.1.2. MRM Transitions and Conditions

Multireaction monitoring (MRM) was chosen for quantification, and the MRM transitions and conditions for GHB, GBL, and 1,4-BD are shown in Table 1.

Although GHB and GBL have the same product ion (*m*/*z* = 45.2) and GBL and 1,4-BD have the same product ion (*m*/*z* 43.2), they have different precursor ions; therefore, the same product ion does not influence their quantification results (Table 1).

#### 3.1.3. Chromatographic Separation

**Figure 4:**
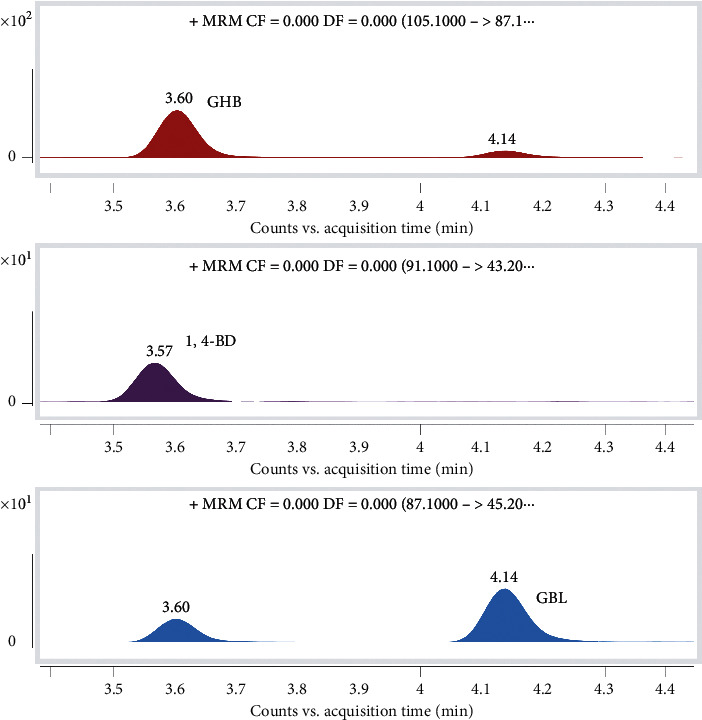
The chromatographic separation results obtained for GBL, GHB, and 1,4-BD are as follows: Rt_GHB_ = 3.60 min, Rt_1,4-BD_ = 3.57 min, and Rt_GBL_ = 4.14 min.

A 5% aqueous solution of methanol was used as the mobile phase in order to obtain the improved chromatographic separation of these compounds due to the high polarity of GHB and 1,4-BD. GHB is a weak acid, GBL is relatively stable, and 1,4-BD exhibits a good response under an acidic environment, and thereby 0.2% formic acid (*v*/*v*) was added into the mobile phase in order to obtain an improvement in the chromatographic separation and mass detection sensitivity. The retention times observed for the three compounds were different, which further reduced the possibility of affecting the quantitative results. The chromatographic separation results are shown in Figure 4.

### 3.2. Method Valuation

#### 3.2.1. Linearity and Measurement Limits

The performance of the developed method was evaluated. The linearity and correlation coefficients (*R*^2^) obtained for GBL, GHB, and 1,4-BD are listed in Table 2. The limit of detection (LOD) and limit of quantification (LOQ) in the different beverages were defined as the concentration giving an *S*/*N* of 3 and 10, respectively, as shown in Table 2.

Table 2 shows that the linearity in the concentration range of 0.2–50 *μ*g/mL was sufficient for quantitative determination. The LOD and LOQ values obtained using the current methods were satisfactory to quantify the three substances in four kinds of beverages.

#### 3.2.2. Matrix Effect

**Figure 5:**
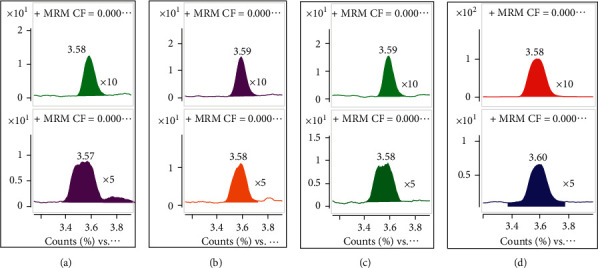
The EICs of the MRM transitions from 105.1 to 87.1 obtained from (a) tea, (b) apple cider vinegar, (c) coffee, and (d) carbonated drinks.

The matrix effect is the main factor influencing the final quantitative results. For the determination of substances in beverages, dilution is the most commonly used method to reduce matrix interference. Two conditions were set to study the influence of the matrix effect, the beverages were diluted 5- and 10-fold, respectively, and then, the working solution was added to make the concentration of these two groups of samples identical. These samples were analyzed using mass spectrometry. The EICs of the MRM transitions from 107.1 to 87.1 were chosen as examples and shown in Figure 5. The upper figures are the EICs of the samples diluted 10-fold and the lower figures are the EICs of the samples diluted 5-fold.

From Figure 5, the signals of the samples diluted 10-fold were much better than those diluted 5-fold. The signals obtained for the samples prepared with a low dilution factor were greatly affected by the matrix; the peak areas of these samples were less than those observed for the samples prepared with a high dilution factor. In addition, the symmetry of the peaks was poor. On the other hand, the signals of the samples prepared with a high dilution factor were slightly affected by the matrix; the peak area and symmetry were similar to those determined in methanol. These results indicate that the matrix effect was negligible when the beverage samples were diluted 10-fold.

#### 3.2.3. Accuracy and Repeatability

**Figure 6:**
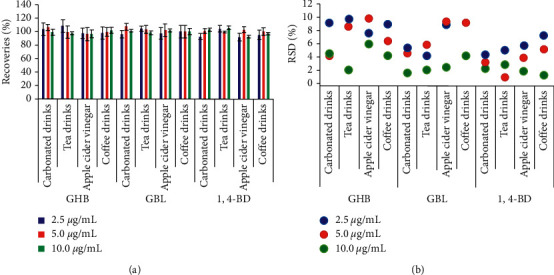
The recoveries and RSDs obtained for GHB, GBL, and 1,4-BD in four kinds of drinks at three spiked levels.

To verify the accuracy of the established method, samples containing GHB, GBL, and 1,4-BD at three spiked levels were prepared at concentrations of 2.5, 5.0, and 10.0 *μ*g/mL. Five copies of each sample were prepared in parallel to examine the repeatability. The results are shown in Figure 6.

Figure 6 shows that the recoveries obtained for GHB, GBL, and 1,4-BD at three spiked levels were between 90 and 110%, which demonstrates that the method has an excellent accuracy. The RSDs of five copied samples at one concentration were all <10%, which indicates that the method has remarkable repeatability.

## 4. Conclusions

This established UHPLC-MS/MS method provides procedures for the identification of multiple drugs of abuse in beverages with fast analysis times. The sensitivity levels required were met, and multiple reaction monitoring of several fragmentation transitions was carried out not only for quantification using the designated quantifying ions but also for confirmation using the designated qualifier ions. This method is a robust tool for the simultaneous determination of illegally added GHB and its precursors (GBL and 1,4-BD) in beverages with excellent repeatability, good reliability, and high sensitivity, which can be used for quality control in the food industry.

## Figures and Tables

**Table 1:** MRM transitions and conditions for GHB, GBL, and 1,4-BD.

Compound	Precursor ion	Product ion	Fragment (V)	CE (V)	Rt (min)
GHB	105.1	87.1	50	2	3.25
		45.2	50	22	
GBL	87.1	45.2	69	14	3.30
		43.2	69	10	
1,4-BD	91.1	73.1	40	2	3.82
		43.2	40	14	

**Table 2:** The linearity, LOD, and LOQ obtained for GHB, GBL, and 1,4-BD.

Compound	Linear equation	*R* ^2^	LOD (*μ*g/mL)	LOQ (*μ*g/mL)
GHB	*y* = 5052.1*x* + 72524	0.9991	0.2	0.5
GBL	*y* = 2192*x* + 117649	0.9992	1	2.5
1,4-BD	*y* = 12856*x* + 22234	0.9995	0.2	0.5

## Data Availability

The data used to support this study are available from the corresponding author upon request.

## Abbreviation

GHB: *γ*-Hydroxybutyrate
GBL: *γ*–Butyrolactone
1,4-BD: 1,4-Butanediol
RSD: Relative standard deviation
GC-MS: Gas chromatography-tandem mass spectrometry
LC-MS: Liquid chromatography-tandem mass spectrometry
UHPLC: Ultrahigh-performance liquid chromatography
MRM: Multireaction monitoring.

## References

[1] Rd S. O., Gibson K. M. (2005). Gamma-hydroxybutyric acid. *New England Journal of Medicine*.

[2] Gallimberti L., Ferri M., Ferrara S. D., Fadda F., Gessa G. L. (2010). Gamma-hydroxybutyric acid in the treatment of alcohol dependence: a double-blind study. *Alcoholism Clinical & Experimental Research*.

[3] Bertol E., Mari F., Vaiano F. (2015). Determination of GHB in human hair by HPLC–MS/MS: development and validation of a method and application to a study group and three possible single exposure cases. *Drug Testing and Analysis*.

[4] Wood D. M., Brailsford A. D., Dargan P. I. (2011). Acute toxicity and withdrawal syndromes related to gamma-hydroxybutyrate (GHB) and its analogues gamma-butyrolactone (GBL) and 1,4-butanediol (1,4-BD). *Drug Testing and Analysis*.

[5] Brunt T., Amsterdam J., Brink W. (2014). GHB, GBL, and 1,4-BD addiction. *Current Pharmaceutical Design*.

[6] Ciolino L. A., Mesmer M. Z., Satzger R. D., Machal A. C., Mccauley H. A., Mohrhaus A. S. (2001). The chemical interconversion of GHB and GBL: forensic issues and implications. *Journal of Forensic Sciences*.

[7] Mehling L.-M., Wang X., Johansen S.-S. (2017). Determination of GHB and GHB-*β*-O-glucuronide in hair of three narcoleptic patients-comparison between single and chronic GHB exposure. *Forensic Science International*.

[8] Elliott S., Burgess V. (2005). The presence of gamma-hydroxybutyric acid (GHB) and gamma-butyrolactone (GBL) in alcoholic and non-alcoholic beverages. *Forensic Science International*.

[9] Kang S., Oh S. M., Chung K. H., Lee S. (2014). A surrogate analyte-based LC–MS/MS method for the determination of *γ*-hydroxybutyrate (GHB) in human urine and variation of endogenous urinary concentrations of GHB. *Journal of Pharmaceutical and Biomedical Analysis*.

[10] Alston W. C., Ng K. (2002). Rapid colorimetric screening test for gamma-hydroxybutyric acid (liquid *X*) in human urine. *Forensic Science International*.

[11] Rosi L., Frediani P., Bartolucci G. (2013). Determination of GHB and its precursors (GBL and 1,4-BD) in dietary supplements through the synthesis of their isotopologues and analysis by the GC–MS method. *Journal of Pharmaceutical and Biomedical Analysis*.

[12] Zacharis C. K., Raikos N., Giouvalakis N., Tsoukali-Papadopoulou H., Theodoridis G. A. (2008). A new method for the HPLC determination of gamma-hydroxybutyric acid (GHB) following derivatization with a coumarin analogue and fluorescence detection. *Talanta*.

[13] Mesmer M. Z., Satzger R. D. (1998). Determination of gamma-hydroxybutyrate (GHB) and gamma-butyrolactone (GBL) by HPLC/UV-VIS spectrophotometry and HPLC/thermospray mass spectrometry. *Journal of Forensic Sciences*.

[14] Dahl S. R., Olsen K. M., Strand D. H. (2012). Determination of gamma-hydroxybutyrate (GHB), beta-hydroxybutyrate (BHB), pregabalin, 1,4-butane-diol (1,4-BD) and gamma-butyrolactone (GBL) in whole blood and urine samples by UPLC–MSMS. *Journal of Chromatography B*.

[15] Sørensen L. K., Rittig N. F., Holmquist E. F. (2013). Simultaneous determination of Β-hydroxybutyrate and Β-hydroxy-Β-methylbutyrate in human whole blood using hydrophilic interaction liquid chromatography electrospray tandem mass spectrometry. *Clinical Biochemistry*.

[16] Sã Rensen L. K., Hasselstrã J. B. (2012). A hydrophilic interaction liquid chromatography electrospray tandem mass spectrometry method for the simultaneous determination of Γ-hydroxybutyrate and its precursors in forensic whole blood. *Forensic Science International*.

[17] Johansen S. S., Windberg C. N. (2011). Simultaneous determination of *γ*-hydroxybutyrate (GHB) and its analogues (GBL, 1.4-BD, GVL) in whole blood and urine by liquid chromatography coupled to tandem mass spectrometry. *Journal of Analytical Toxicology*.

[18] Wood M., Laloup M., Samyn N. (2004). Simultaneous analysis of gamma-hydroxybutyric acid and its precursors in urine using liquid chromatography–tandem mass spectrometry. *Journal of Chromatography A*.

[19] Dasenaki M., Thomaidis N. (2019). Quality and authenticity control of fruit juices-A review. *Molecules*.

[20] Thomsen R., Rasmussen B. S., Johansen S. S., Linnet K. (2017). Postmortem concentrations of gamma-hydroxybutyrate (GHB) in peripheral blood and brain tissue—differentiating between postmortem formation and antemortem intake. *Forensic Science International*.

[21] Klupczynska A., Plewa S., Dyszkiewicz W., Kasprzyk M., Sytek N., Kokot Z. J. (2016). Determination of low-molecular-weight organic acids in non-small cell lung cancer with a new liquid chromatography–tandem mass spectrometry method. *Journal of Pharmaceutical and Biomedical Analysis*.

[22] Hanisch S., Stachel N., Skopp G. (2016). A potential new metabolite of gamma-hydroxybutyrate: sulfonated gamma-hydroxybutyric acid. *International Journal of Legal Medicine*.

[23] Remane D., Wetzel D., Peters F. T. (2014). Development and validation of a liquid chromatography–tandem mass spectrometry (LC–MS/MS) procedure for screening of urine specimens for 100 analytes relevant in drug-facilitated crime (DFC). *Analytical and Bioanalytical Chemistry*.

[24] Dziadosz M., Weller J.-P., Klintschar M., Teske J. (2013). Adduct supported analysis of *γ*-hydroxybutyrate in human serum with LC–MS/MS. *Analytical and Bioanalytical Chemistry*.

